# Supplementary material for: Franz Josef Land: extreme northern outpost for Arctic fishes
Source: PeerJ. 2014 Dec 11;2:e692. doi: 10.7717/peerj.692 (PMC4266852; doi:10.7717/peerj.692)
Supplement: Table S1 [file peerj-02-692-s001.docx]

| No. | Scientific name | English name | Nearshore  (≤ 34 m) | Offshore  (m) | References |
| --- | --- | --- | --- | --- | --- |
|  | SQUALIFORMES |  |  |  |  |
|  | 1.Somniosidae D.S. Jordan, 1888 | Sleeper Sharks |  |  |  |
| 1 | *Somniosus microcephalus* (Bloch et Schneider, 1801) | Greenland shark | 211* | - | This expedition |
|  | RAJIFORMES |  |  |  |  |
|  | 2. Rajidae Bonaparte, 1831 | Skates |  |  |  |
| 2 | *Amblyraja hyperborea* (Collett 1879) | Arctic skate | - | 210-590 | Borkin, 1993; Wienerroither et al., 2011 |
| 3 | *Amblyraja radiata* (Donovan, 1808) | Thorny ray | - | + | Dolgov, 2011 |
|  | osmeriformes |  |  |  |  |
|  | 3. Osmeridae Regan, 1913 | Smelts |  |  |  |
| 4 | *Mallotus villosus* (Müller, 1776) | Capelin | - | + | Borkin, 1993; Wienerroither et al., 2011 |
|  | AULOPIFORMES |  |  |  |  |
|  | 4. Paralepididae Bonaparte, 1839 |  |  |  |  |
| 5 | *Arctozenus rissoi* (Bonaparte, 1840) | White Barracudina | - | + | Wienerroither et al., 2011 |
|  | MYCTOPHIFORMES |  |  |  |  |
|  | 5. Myctophidae Th.N. Gill, 1893 | Lanternfishes |  |  |  |
| 6 | *Benthosema glaciale* (Reinhardt, 1837) | Glacier Lanternfish | - | 310-340 | Borkin, 1986; Borkin, 1993; Wienerroither et al., 2011 |
|  | gadiformes |  |  |  |  |
|  | 6. Gadidae Rafinesque, 1810 | Codfishes |  |  |  |
| 7 | *Arctogadus glacialis* (Peters, 1874) | Arctic or Black cod | - | 230-330 | Borkin, 1993; Wienerroither et al., 2011 |
| 8 | *Boreogadus saida* (Lepechin, 1774) | Polar cod | 6-21 | 170-460 | Knipowitch, 1901; Camerano, 1903; Esipov, 1931; Burmakin, 1957; Andriashev, 1964; Borkin, 1993; Wienerroither et al., 2011; This expedition |

| No. | *Scientific name* | English name | Nearshore FJL  (≤ 34 m) | Offshore FJL  (m) | References |
| --- | --- | --- | --- | --- | --- |
| 9 | *Gadus morhua* Linnaeus, 1758 | Atlantic cod | 15 | + | Borkin, 1993; Wienerroither et al., 2011; This expedition |
|  | SCORPAENIFORMES |  |  |  |  |
|  | 7. Scorpaenidae A. Risso, 1827 | Scorpionfishes |  |  |  |
| 10 | *Sebastes mentella* Travin, 1951 | Beaked redfish | - | 370-590 | Borkin, 1993; Wienerroither et al., 2011 |
|  | 8. Cottidae Bonaparte, 1831 | Sculpins |  |  |  |
| 11 | *Artediellus europaeus* Knipowitsch, 1907 | European hookear sculpin | - | 230-410 | Borkin, 1993; Wienerroither et al., 2011 |
| 12 | *Artediellus scaber* Knipowitsch, 1907 | Hamecon | 6-21 | 204-370 | Knipowitch, 1901; Borkin, 1993, This expedition |
| 13 | *Icelus bicornis* (Reinhardt, 1840) | Twohorn sculpin | 7-21 | 26-110 | Knipowitch, 1901; Esipov, 1931; Andriashev, 1964; Borkin, 1993, This expedition |
| 14 | *Triglops murrayi* Günther, 1888 **** | Moustache sculpin | - | + | Wienerroither et al., 2011 |
| 15 | *Triglops nybelini* Jensen, 1944 | Bigeye sculpin | + | 124-510 | Knipowitch, 1901; Andriashev, 1964; Borkin, 1993; This expedition |
| 16 | *Triglops pingelii* Reinhardt, 1831 **** | Ribbed sculpin | - | + | Wienerroither et al., 2011 |
|  | 9. Agonidae Swainson, 1839 | Sea Poachers |  |  |  |
| 17 | *Leptagonus decagonus* (Bloch et Schneider, 1801) | Atlantic poacher | 10 | 245-400 | Borkin, 1993; Wienerroither et al., 2011; This expedition |
|  | 10. Psychrolutidae Günther, 1861 | Fatheads |  |  |  |
| 18 | *Cottunculus microps* Collett, 1875 | Polar fathead | - | 245-590 | Borkin, 1993; Wienerroither et al., 2011 |
| 19 | *Cottunculus sadko* Essipov, 1937 | Sadko fathead | - | 330-460 | Borkin, 1993 |
|  | 11. Cyclopteridae Bonaparte, 1831 | Lumpfishes |  |  |  |
| 20 | *Cyclopteropsis* *mcalpini* (Fowler, 1914) | McAlpin’s Smooth lumpfish | 18-31 | 124 | Andriashev, 1964; Borkin, 1993; This expedition |

| No. | Scientific name | English name | Nearshore FJL  (≤ 34 m) | Offshore FJL  (m) | References |
| --- | --- | --- | --- | --- | --- |
| 21 | *Eumicrotremus derjugini* Popov, 1926 | Derjugin’s Leatherfin Lumpsucker | 6-21 | 240 | Borkin, 1993; Wienerroither et al., 2011 |
| 22 | *Eumicrotremus spinosus* (Fabricius, 1776) | Atlantic spiny lumpsucker | 8-15 | 34 | Knipowitch, 1901; Borkin, 1993; Wienerroither et al., 2011; This expedition |
|  | 12. Liparidae Th.N. Gill, 1861 | Snailfishes |  |  |  |
| 23 | *Liparis bathyarcticus* Parr, 1931 | Parr snailfish | 6-15 | 280-460 | Chernova, 1991 (as *L. gibbus*); Borkin, 1993 (as *L. gibbus*) ; Wienerroither et al., 2011; This expedition |
| 24 | *Liparis* сf. *fabricii* Krøyer, 1847 | Blackbelly snailfish | 10-25 | 110-392 | Knipowitch, 1901; Andriashev, 1964 (as *L. koefoedi*); Chernova, 1991, 1993; Borkin, 1993; Wienerroither et al., 2011; This expedition |
| 25 | *Liparis tunicatus* Reinhardt, 1837 | Kelp snailfish | 6-34 | - | Knipowitch, 1901 (as *Cyclogaster liparis*); Esipov, 1933 (as *L. liparis*); Chernova, 1991; This expedition |
| 26 | *Careproctus* cf. *micropus* (Günther, 1887) | Smalleyed tadpole | - |  | Chernova, 1991 |
| 27 | *Careproctus* cf. *ranula* (Goode et Bean, 1880) | Smallhead tadpole | - |  | Chernova, 1991 |
| 28 | *Сareproctus* cf. *reinhardti* (Krøyer, 1862) | Reinhardt’s tadpole | - |  | Knipowitch, 1901 (as *Cyclogaster gelatinosus*); Chernova, 1991 |
|  | PERCIFORMES |  |  |  |  |
|  | 13. Zoarcidae Swainson, 1839 | Eelpouts |  |  |  |
| 29 | Lycodes esmarkii Collett, 1875 | Esmark's ellpout | - | + | Wienerroither et al., 2011 |

| No. | *Scientific name* | English name | Nearshore FJL  (≤ 34 m) | Offshore FJL  (m) | References |
| --- | --- | --- | --- | --- | --- |
| 30 | *Lycodes eudipleurostictus* Jensen, 1902 | Doubleline eelpout | - | 185-460 | Andriashev, 1964; Borkin, 1993; Wienerroither et al., 2011 |
| 31 | *Lycodes pallidus* Collett, 1879 | Pale eelpout | - | 180-410 | Andriashev, 1964; Borkin, 1993; Wienerroither et al., 2011 |
| 32 | *Lycodes polaris* (Sabine, 1824) | Polar eelpout | - | 210-590 | Borkin, 1993; Wienerroither et al., 2011 |
| 33 | *Lycodes reticulatus* Reinhardt, 1835 | Arctic eelpout | 6-15 | 124-410 | Andriashev, 1964; Borkin, 1993; Wienerroither et al., 2011; This expedition |
| 34 | *Lycodes rossi* Malmgren, 1865 | Threespot eelpout | - | 230-400 | Andriashev, 1964; Borkin, 1993; Wienerroither et al., 2011 |
| 35 | *Lycodes seminudus* Reinhardt, 1837 | Halfnaked Eelpout | - | 250-410 | Borkin, 1993; Wienerroither et al., 2011 |
| 36 | *Gymnelus andersoni* Chernova, 1998 | Anderson’s pout | + | + | Borkin, 1993 (as *G. retrodorsalis*), Chernova, 1998а; This expedition |
| 37 | *Gymnelus esipovi* Chernova, 1999 | Esipov’s pout | + | - | This expedition |
| 38 | *Gymnelus taeniatus* Сhernova, 1999 | Tapebody pout | + | - | Chernova, 1999а |
|  | 14. Lumpenidae |  |  |  |  |
| 39 | *Anisarchus medius* (Reinhardt, 1837) | Stout eelblenny | - | 170-190 | Borkin, 1993 |
| 40 | *Leptoclinus maculatus* (Fries, 1837) | Spotted Snake Blenny | - | - | Borkin, 1993 |
|  | PLEURONECTIFORMES |  |  |  |  |
|  | 15. Pleuronectidae Rafinesque, 1815 | Righteyed Flounders |  |  |  |
| 41 | *Glyptocephalus cynoglossus* (Linnaeus, 1758) | Witch flounder | - | 410 | Borkin, 1993 |
| 42 | *Hippoglossoides platessoides limandoides* (Bloch, 1787) | Long rough dab | - | 270-570 | Borkin, 1993; Wienerroither et al., 2011 |

| No. | *Scientific name* | English name | Nearshore FJL  (≤ 34 m) | Offshore FJL  (m) | References |
| --- | --- | --- | --- | --- | --- |
| 43 | *Reinhardtius hippoglossoides* (Walbaum, 1792) | Greenland halibut | - | 210-620 | Borkin, 1993; Wienerroither et al., 2011 |

* recorded in a strait near Hayes island; ** - identification need to be checked.
